# Supplementary material for: Mortality and comorbidities among teaching professionals: A cross-sectional study in Colombia
Source: PLoS One. 2026 Jan 6;21(1):e0332110. doi: 10.1371/journal.pone.0332110 (PMC12774334; doi:10.1371/journal.pone.0332110)
Supplement: S1 File — (DOCX) [file pone.0332110.s001.docx]

**Supplementary File 1. Teaching Activity Codes**

| **Teaching activity** | **Codes related** |
| --- | --- |
| Preschool | 8511, 8512 |
| Primary education | 8513 |
| Lower secondary education | 8521 |
| Upper secondary education | 8522 |
| Technical/technological education | 8541, 8542 |
| Universities | 8543 |
| Other education sectors | 6495, 8530, 8552, 8553, 8559 |
