# Supplementary material for: Mortality and comorbidities among teaching professionals: A cross-sectional study in Colombia
Source: PLoS One. 2026 Jan 6;21(1):e0332110. doi: 10.1371/journal.pone.0332110 (PMC12774334; doi:10.1371/journal.pone.0332110)
Supplement: S2 File — (PDF) [file pone.0332110.s002.pdf]

Translated version

Official Reference
Reference No.: 201913000267461
Date: 05 March 2019

Bogotá D.C., Colombia

Doctor
GIANCARLO BUITRAGO
Associate Professor, Department of Surgery
Institute of Clinical Research
Universidad Nacional de Colombia
Bogotá D.C., Colombia

SUBJECT: Request for health information – Reference No. 201842301411402

Dear Sir,
In response to your request for anonymised information from the health data sources of interest, we hereby inform you of the following:

1. For the dataset corresponding to health insurance affiliation records, PILA, Health Services Information – Sufficiency Study, and Births/Deaths (RUAF-ND) for the period 2010–2016, the Department of Clinical Epidemiology and Biostatistics is authorised to deliver this dataset (authorisation letter attached, Ref. 201913000267381) to the Institute of Clinical Research of the Universidad Nacional de Colombia.

2. For the MIPRES dataset, variables of interest must be defined in accordance with Resolution 1885 of 2018.

3. The datasets corresponding to health insurance affiliation records, PILA, and Health Services Information – Sufficiency Study for the year 2017, as well as RIPS data for the period 2011–2017, are currently being processed. These datasets will be delivered gradually due to the processing time required and the volume of information involved. As the files become available, you will be informed so that an external storage device can be provided for the corresponding data transfer.

This information is delivered in accordance with Law 1581 of 2012, which establishes general provisions for the protection of personal data, and its use must guarantee the right to habeas data. It is important to note that the Constitutional Court, in Ruling C-748/2011, states that once an administrative entity accesses personal data, it assumes the legal position of a data user within the personal data management process, which logically imposes upon it the duty to guarantee the fundamental rights of the data subject, as established in the Political Constitution, and consequently requires that they:

(i) *Maintain the confidentiality of the information supplied by the operators and use it exclusively for the purposes that justified its delivery, namely those related to the specific functional competence that motivated the request for the provision of personal data;*
(ii) *Inform the data subjects of the use being made of their data;*
(iii) *Safeguard the received information with appropriate security measures in order to prevent its deterioration, loss, alteration, unauthorised or fraudulent use; and*

(iv) *Comply with the instructions issued by the supervisory authority regarding compliance with statutory legislation.*

This Office requests that the existence of these datasets be disseminated within the University, with the aim of facilitating access for interested parties and avoiding reprocessing of the same information at the Ministry level, thereby optimising resources.

Finally, it is important to emphasise that for the Ministry of Health and Social Protection, and particularly for this Office, it would be of great benefit to be informed of the results of the research projects you undertake using the delivered data. We therefore look forward to being informed of the outcomes of such projects.

Sincerely,

DOLLY ESPERANZA OVALLE CARRANZA
Head of the Office of Information and Communication Technology
Ministry of Health and Social Protection

La salud es de todos

Minsalud

201913000267461

Radicado No.: **201913000267461**

Fecha: **05-03-2019**

Bogotá D.C.,

Doctor
GIANCARLO BUITRAGO
Profesor asociado Departamento de Cirugía
Instituto de Investigaciones Clínicas
Universidad Nacional de Colombia
gbuitragog@unal.edu.co
No registra
Bogotá D.C.

ASUNTO: Solicitud información salud, Rad.201842301411402

Cordial saludo.

En atención a su solicitud de información anonimizada para las fuentes de interés, nos permitimos informarle:

1. Para el conjunto de datos de: Afiliados a salud, PILA, Información de Servicios de Salud – Estudio de Suficiencia y Nacimientos/Defunciones – RUAF-ND del período 2010-2016, se autoriza al Departamento de Epidemiología Clínica y Bioestadística a realizar la entrega de este conjunto de datos (adjunto oficio de autorización, rad.201913000267381) al Instituto de Investigaciones Clínicas de la Universidad Nacional.

2. Para el conjunto de datos de MIPRES, es necesario que definan las variables de interés, para lo cual se sugiere revisar la Resolución 1885 de 2018.

3. El conjunto de datos de Afiliados a salud, PILA e Información de Servicios de Salud – Estudio de Suficiencia del año 2017 y RIPS 2011-2017 están siendo procesados y se realizarán entregas graduales, debido al tiempo de procesamiento que demandan y volumen que ocupan. En la medida que se tengan disponibles los archivos se les informará para que dispongan del dispositivo externo para su respectiva copia.

Se hace entrega de esta información en los términos de la Ley 1581 de 2012 – *por el cual se dictan disposiciones generales para la protección de datos personales* – y su uso debe garantizar el derecho de habeas data. Es importante tener en cuenta la Corte Constitucional en Sentencia C-748/2011, señala que una vez la entidad administrativa

accede al dato personal adopta la posición jurídica de usuario dentro del proceso de administración de datos personales, lo que de forma lógica le impone el deber de garantizar los derechos fundamentales del titular de la información, previstos en la Constitución Política y en consecuencia deberán:

(i) " guardar reserva de la información que les sea suministradora por los operadores y utilizarla únicamente para los fines que justificaron la entrega, esto es, aquellos relacionados con la competencia funcional específica que motivó la solicitud de suministro del dato personal;
(ii) informar a los titulares del dato el uso que le esté dando al mismo;
(iii) conservar con las debidas seguridades la información recibida para impedir su deterioro, pérdida, alternación, uso no autorizado o fraudulento; y
(iv) cumplir con las instrucciones que imparta la autoridad de control, en relación con el cumplimiento de la legislación estatutaria".

Por parte de esta Oficina se solicita socializar la existencia de estos datos al interior de la Universidad, en aras a facilitar su consulta por parte de los interesados y evitar reprocesos a nivel del Ministerio de esta misma información, optimizando de esta manera los recursos.

Finalmente, es importante resaltar que para el Ministerio de Salud y Protección Social y en particular para esta Oficina será de gran beneficio conocer los resultados de los proyectos de investigación que ustedes adelanten utilizando los datos entregados, por lo que estaremos atentos a conocer el resultado de esos proyectos.

Atentamente,

DOLLY ESPERANZA OVALLE CARRANZA
Jefe Oficina de Tecnología de la información y la Comunicación

Adjunto: Oficio autorización entrega Universidad Pontificia Universidad Javeriana

Elaboró: LuzR / Revisó/Aprobó: MaríaE
C:\LRincon\Trabajo LRINCONM\MINSALUD 2012\ORFEO 2019\Usuarios Externos
