# Supplementary material for: Mortality and comorbidities among teaching professionals: A cross-sectional study in Colombia
Source: PLoS One. 2026 Jan 6;21(1):e0332110. doi: 10.1371/journal.pone.0332110 (PMC12774334; doi:10.1371/journal.pone.0332110)
Supplement: Attachment — Submitted filename: Reviewers comments.docx [file pone.0332110.s004.docx]

**Reviewer’s Report**

**Manuscript Title:**
*Overall Mortality and Comorbidities Associated with the Teaching Profession: A Cross-Sectional Study in Colombia*

**General Comments:**
The study addresses an important public health concern by examining mortality patterns and comorbidities among teachers in Colombia. The topic is relevant and potentially contributes to understanding occupational health risks in the education sector. However, the manuscript requires revisions to improve clarity, organization, and scholarly rigor.

**Specific Comments**

1. **Title Revision**
   The title should be rephrased for better clarity and precision. The current version can be refined as follows:
   *“Mortality and Comorbidities among Teaching Professionals: A Cross-Sectional Study in Colombia.”*
   This rephrasing maintains the original meaning while improving readability and academic tone.
2. **Keywords**
   A few more relevant keywords should be included to enhance searchability and indexing. In addition to existing ones, consider adding terms such as *occupational health, teachers, mortality rate, comorbidity patterns,* and *Colombian educators.*
3. **Introduction and Literature Review**
   The literature review needs to be extended. At present, it appears too brief and insufficiently contextualized. The introduction should include a more comprehensive discussion of prior studies related to occupational health risks, mortality, and comorbidities in the teaching profession—both globally and regionally. This will help establish the research gap and strengthen the rationale for the study.
4. **Ethical Approval Statement**
   The ethical approval statement should not be included within the methodology section. It should be placed separately under a distinct subheading, such as *“Ethical Considerations”* or *“Ethical Approval,”* following standard research reporting conventions.
5. **Data Transparency and Verification**
   Since the study utilizes four primary administrative databases anonymized and linked using 188 unique identifiers by the Ministry of Health (MoH), a **hyperlink** or citation should be provided to allow readers to verify the authenticity and source of these datasets. This step will enhance transparency and credibility.

The manuscript has potential for publication after **major revisions**. Addressing the above issues will significantly improve the clarity, scholarly depth, and transparency of the study.
